# Antimicrobial coatings effectively inactivate multidrug-resistant *Candidozyma auris* on surfaces

**DOI:** 10.3389/fmicb.2025.1666364

**Published:** 2025-10-20

**Authors:** Sabine Poelzl, Eva Zarschenas, Rozita Nokhbehzaeim, Kathrin Spettel, Clemens Kittinger

**Affiliations:** ^1^Diagnostic and Research Institute for Hygiene, Microbiology and Environmental Medicine, Medical University of Graz, Graz, Austria; ^2^Division of Clinical Microbiology, Department of Laboratory Medicine, Medical University of Vienna, Vienna, Austria; ^3^Department of Biomedical Science, Health Sciences, University of Applied Sciences Campus Vienna, Vienna, Austria

**Keywords:** *Candidozyma auris*, *Candida albicans*, antimicrobial surface, antifungal, ISO 22196:2011, multidrug-resistant, hygiene measures

## Abstract

*Candidozyma auris*—formerly known as *Candida auris*—is an emerging multidrug-resistant fungus causing life-threatening outbreaks, particularly in healthcare settings. Its ability to contaminate hospital equipment, persist on certain surfaces and develop resistance to commonly used antifungal agents makes it a critical priority pathogen. While classical hygiene measures are essential, increasing resistance to disinfectants calls for alternative approaches. One promising strategy is the use of antimicrobial materials on frequently touched surfaces to minimize the survival rate of problematic microorganisms. As literature does not provide a lot of data regarding the survival of *Candida* genera on surfaces, the present study was undertaken to analyze the survival/elimination of *C. auris* on different common and specific surfaces. ISO 22196:2011 was used to generate an overview of the efficacy of these surfaces against DSMZ strains of *C. auris* and *C. albicans*, respectively. The findings indicate that *C. albicans* can be regarded as suitable model organism for *C. auris*. Three clinical *C. auris* isolates with different genetic characteristics and/or phylogeographic origins yielded similar results to the DSMZ strains, providing a clear indication of the antifungal efficacy of surfaces tested. While reference materials without antimicrobial additives showed no efficacy, a combination of zinc and copper achieved the required 3 log_10_ reduction after 24 h. Most effective against all fungal strains in two different types of tests was a layer of antimicrobial lacquer, which showed a significant decrease in fungal survival within 1 h. Thus, these surface modifications can be considered as effective tools for fighting *C. auris* in hygienically critical areas.

## 1 Introduction

Human fungal infections have historically been a neglected area of research, despite the fact that over six million people are affected by an immediately life-threatening fungal disease each year (Denning, 2024). Research is now focused on understanding and targeting the pathogenesis of fungal infections (Brown et al., 2024) including those caused by the emerging multidrug-resistant fungus *Candidozyma auris* (*C. auris*). This fungal pathogen was isolated for the first time in 2009 from the discharge of the external auditory canal of a 70-year-old Japanese patient (Satoh et al., 2009). This has led to the potentially misleading name “*auris”* which derives from the Latin word for “ear.” However, this fungus is not only associated with otitis, but also with invasive fungal infections (IFIs) in vulnerable patients and was frequently detected in the bloodstream, wounds, catheter tips and intra-abdominal infections (De Gaetano et al., 2024). Since its first appearance, it emerged simultaneously on different continents (Lockhart et al., 2017). Over 2,500 cases have been reported in Africa alone by 2023 (Osaigbovo et al., 2024) with almost 100 affected hospitals in the south of the continent (De Gaetano et al., 2024). Recently, an increase in the number of cases has also been observed in Europe and the US. It is therefore regarded as an urgent threat by US Centers for Disease Control and Prevention (five-fold increase in reported clinical cases from 2019 to 2022) (Centers for Disease Control and Prevention, 2022) and was classified in the Critical-priority group by WHO (Casalini et al., 2024). The greatest risk is given in healthcare settings, where it can easily spread between immunocompromised patients through contaminated surfaces or objects with high case fatality rates for invasive infections (De Gaetano et al., 2024). In addition, healthy people are usually unaffected, but healthcare workers in particular are a source of transmission as *C. auris* can persist asymptomatically on the skin. This can lead to major outbreaks in healthcare settings as shown by the example of the first outbreak in Europe. In this case, 50 *C. auris* infections were reported at a single hospital in London between April 2015 and July 2016. Outbreak investigations have shown that the innate resilience of this pathogen to higher temperatures or high salt concentrations (De Gaetano et al., 2024) in terms of survival and persistence in the clinical setting, its ability to rapidly colonize the skin of patients and its high transmissibility in healthcare settings led to this severe and prolonged outbreak (Schelenz et al., 2016).

*C. auris* cases have also been detected in Austria in recent years. Five sporadic cases, including one infection and four cases of asymptomatic colonization, have already been published from different regions of Austria. They were further analyzed and characterized as South Asian clade I or as African clade III with aggregative or non-aggregative growth phenotypes. Furthermore, it was observed that all of these five clinical isolates exhibited elevated minimal inhibitory concentration (MIC) values to at least two distinct classes of antifungals (Spettel et al., 2023). However, it is conceivable that more cases of *C. auris* occurred in Austria without being reported yet. In addition, commercial identification systems (e.g., biochemical methods) often result in misidentification. The utilization of advanced methodologies such as MALDI-TOF MS or DNA-based methods, is strongly advocated for the identification of *C. auris* (De Gaetano et al., 2024) and thereby facilitating the implementation of infection control measures in a timely and effective manner.

Another concern is the increasing antifungal drug resistance against commonly used therapeutics, including the first-line therapy for the most invasive *Candida* infections, echinocandin. The combination of this resistance with resistance to azole and amphotericin B is considered as a significant clinical and public health concern. Approximately 50% of *C. auris* isolates from five continents are estimated to be resistant to one or more classes of antifungal agents (Lone and Ahmad, 2019; De Gaetano et al., 2024). Therefore, different classical hygiene strategies to eliminate *C. auris* have already been investigated, including disinfectants and antiseptics (e.g., hydrogen peroxide, ethanol, chlorhexidine gluconate or sodium hypochlorite), which can reduce ≥4 log_10_ CFU within 1 min or lead to a 100% killing after longer exposure time (Cadnum et al., 2017; Kean et al., 2018; Bandara and Samaranayake, 2022). However, the effectiveness of these products depends on several key factors (Bandara and Samaranayake, 2022). Widely used quaternary ammonium disinfectants, for instance, show only a poor activity against *C. auris* (Cadnum et al., 2017; Kean and Ramage, 2019). Another increasingly used decontamination strategy is ultraviolet (UV) light therapy (Bandara and Samaranayake, 2022; Koutras and Wade, 2024).

Based on the numerous facts that have been enumerated it is evident, that *C. auris* poses a significant burden to the public health system and is likely to result in further hospital outbreaks in the future. Consequently, any measures implemented with the intention of preventing the spread of infection should not be disregarded. *C. auris* has been found to survive on human skin and environmental surfaces for several weeks to months (Ahmad and Alfouzan, 2021). For instance, Welsh et al. described a survival on plastic surfaces of at least 14 days, however, esterase activity can be measured on the same type of surface for up to 1 month (Welsh et al., 2017). Isolates from patients have the ability to contaminate various surfaces, including the immediate environments such as bed rails, mattresses, door handles, flooring, walls and windowsills or can even spread to bathing areas. In addition, medical equipment that comes in contact with the patient can easily become contaminated, facilitating the spread of this fungus to other patients (Chakrabarti and Sood, 2021). In the light of these findings, the application of antimicrobial materials (AMMs) could be a promising additional measure. These materials can be divided into three groups based on their mechanism of action: active substance-release AMMs, potentiated surface-based AMMs (including biocides, metals, peptides or amines on their surfaces), and non-adhesive AMMs (Campoccia et al., 2013; Sjollema et al., 2018; Cunliffe et al., 2021). Many different AMMs have already been tested for their antimicrobial efficacy. However, it should be noted that testing against fungi in this field of science has not been a primary focus yet, as well-known standardized methods currently available only provide either antibacterial (International Organization for Standardization, 2011) or antiviral (International Organization for Standardization, 2016, 2019) assays. Thus, data concerning antifungal surfaces remains limited, especially for *C. auris*. Until now, mainly non-specific surfaces without AMMs, such as glass, steel, wood, ceramic or plastic have been tested to obtain a general survival time (Piedrahita et al., 2017; Welsh et al., 2017; Dire et al., 2023).

The primary objective of this study was to evaluate different common and specific surfaces for their antifungal efficacy and to find a potential AMMs to eliminate *C. auris* quickly. In addition, a comparative analysis was conducted between the DSMZ strains of *C. auris* and *C. albicans* to determine if these two *Candidozyma*/*Candida* species exhibited analogous behavior on the surfaces tested. Finally, three *C. auris* patient isolates were tested on the same surfaces to establish a link to clinically relevant cases.

## 2 Materials and methods

### 2.1 Samples

Different non-porous surfaces were analyzed in this study. Low alloyed carbon steel (LACS) was provided and coated by voestalpine Stahl GmbH (Linz, Austria). Zinc (Zn) was applied either through hot-dip galvanization (HG) or through electrolytic galvanization (EG) by voestalpine Stahl GmbH (Linz, Austria). LACS + Zn (HG) was further processed by G&S Solution (Bruchsal, Germany) through the application of a layer of clear lacquer, both in the absence (TX-7770) and presence of antimicrobial active substances (AMC-TX-7770) The surface properties of the lacquer modifications were smoother than those of the LACS samples (comparable with Glass) and an application of this lacquer is in general possible to different substrates. Further information on the coatings is currently unavailable due to manufacturer confidentiality. LACS + Zn (EG) were further coated with a thin layer (1–5 nm) of copper (Cu) by voestalpine Stahl GmbH (Linz, Austria). Glass (Cloeren Technology GmbH, Wegberg, Germany) served as overall reference material (Table 1). All tested samples had the size of 50 × 50 mm and were stored at room temperature. Prior to testing, the surfaces were sterilized with 70% ethanol (Merck KGaA, Darmstadt, Germany).

**Table 1:** List of samples used in this study.

Name	Substrate	Coating
Glass	Glass	–
LACS + Zn (HG)	Low alloyed carbon steel	Zinc (hot-dip galvanization)
TX-7770	Low alloyed carbon steel	Zinc (hot-dip galvanization) + layer of clear lacquer
AMC-TX-7770	Low alloyed carbon steel	Zinc (hot-dip galvanization) + layer of clear lacquer with antimicrobial active substances
LACS + Zn (EG)	Low alloyed carbon steel	Zinc (electrolytic galvanization)
LACS + Zn (EG) + Cu	Low alloyed carbon steel	Zinc (electrolytic galvanization) + thin layer of copper (1–5 nm)

### 2.2 Strains

For the antimicrobial test, *Candida albicans* (*C. albicans*) DSM 1386 and *Candidozyma auris* (*C. auris*) DSM 21092 were purchased from a collection of the Leibniz Institute DSMZ (German Collection of Microorganisms and Cell Cultures GmbH, Braunschweig, Germany). Three clinical isolates from *C. auris* were provided by colleagues from the Medical University of Vienna (Division of Clinical Microbiology, Department of Laboratory Medicine, Medical University of Vienna, Austria) (Spettel et al., 2023) (Table 2).

**Table 2:** *Candidozyma/Candida* strains used for the antifungal surface tests.

*C. albicans*	*C. auris*
DSM 1386	DSM 21092 (first East Asia clade II)
	Cau1 (South Asian clade I)
	Cau4 (African clade III)
	Cau5 (South Asian clade I)

### 2.3 Testing of antifungal activity

The antimicrobial assay was based on ISO 22196:2011 measurement of antibacterial activity on plastics and other non-porous surfaces with some adaptations (International Organization for Standardization, 2011). The different yeast strains were cultivated on Columbia Blood agar plates (Becton Dickinson Austria GmbH, Vienna, Austria) at 25 °C ± 2 °C for 72 h (*C. auris*) or 30 °C ± 2 °C for 48 h (*C. albicans*). Cell material was inoculated in a 0.2% v/v Tryptone Soy Broth (TSB, Oxoid Ltd., Basingstoke, United Kingdom) diluted in distilled water. A VITEK® DensiCHEK instrument (Biomerièux, Vienna, Austria) was used to obtain a solution with 1 × 10^8^ colony-forming units (CFU)/mL. An amount of 200 μL of fungal suspension with an expected concentration of 2.5 × 10^5^–10 × 10^5^ cells/mL was pipetted on the sterile surfaces of the samples. The suspension was covered with a sterilized 40 × 40 mm polyethylene film (VWR International, Vienna, Austria) and distributed under the film. Subsequently, the inoculated test specimens were incubated in a wet chamber at strain specific temperatures, adapted to information provided by Leibniz Institute DSMZ (*C. auris* at 25 °C ± 2 °C and *C. albicans* at 30 °C ± 2 °C) with a relative humidity (RH) of approximately 96% for 1 hour and 24 h. As a control, the yeast suspension was harvested immediately after pipetting below the foil (0 h) to ensure initial concentration from each specimen. After the exposure treatment, the samples were rinsed off the surface by four washing steps with 10 mL of neutralizer (SCDLP medium) containing TSB (Oxoid Ltd., Basingstoke, United Kingdom), lecithin (Carl Roth GmbH + Co Kg, Karlsruhe, Germany), and Tween®80 (Amresco Inc., Solon, OH, United States) in order to rescue surviving yeasts. The samples were then shaken in the neutralization medium for 3 min at 200 rpm on a Battery Shaker KM 2 Akku (Edmund Bühler GmbH, Bodelshausen, Germany). An amount of 500 μL of appropriate dilutions in phosphate-buffered solution (PBS, Carl Roth GmbH + Co Kg, Karlsruhe, Germany) was plated on tryptic soy agar (TSA, VWR International Ltd., Vienna, Austria) plates in duplicates. CFUs from *C. auris* strains were counted after incubation for 72 h/96 h at 25 °C ± 2 °C, while plates with *C. albicans* were incubated for 48 h at 30 °C ± 2 °C and then evaluated.

The applied load is defined as the actual number of yeast cells applied to the samples in the experiment. Time point 0 h is defined as the test point where the fungal suspension is harvested immediately after pipetting under the foil to ensure initial concentration of each sample. For each incubation time, triplicates were used in two independent runs (*n* = 6) to calculate mean and standard deviation for the antifungal activity of the specimens. When no colonies were countable on the plates, the limit of detection was set as 10 CFU, since 10 mL of the neutralization medium was used.

For each test sample, the recovered number of viable yeasts in CFU was calculated using the following formula from ISO 18071:2016 (International Organization for Standardization, 2016):


N=C∗V∗D

*N*the number of viable yeasts recovered per test specimen [CFU].

*C*average plate count for the duplicate plates.

*V*volume, in mL, of SCDLP added to the specimen (10 mL).

*D*dilution factor for the plates counted.

Note: The calculation was done in CFU to align with the results of the dry test. Comparability with other studies calculating results in CFU/cm^2^ may be slightly reduced (inoculated surface area = 1,600 mm^2^).

The calculation of the reduction compared to 0 h results was done with following formula:

$R=\frac{\left(U_{0}-U_{t}\right)}{U_{0}}$ or $R=\frac{U_{0}}{U_{t.}}$

*R*the antifungal activity [%] or [log_10_].

*U*_0_the average of the common logarithm of the number of viable yeasts recovered from the test specimens immediately after inoculation (0 h).

*U_t_*the average of the common logarithm of the number of viable yeasts recovered from the test specimens after 1 h.

The verification of the methodology was calculated according to the guideline of ISO 22196:2011 (International Organization for Standardization, 2011) through the 0 h triplicates:


$$(L_{\max}-L_{m\text{in}})L_{\text{mean}}\leq 0.2$$

*L*_max_10 logarithm of the maximum number of viable yeasts found on a specimen.

*L*_min_10 logarithm of the minimum number of viable yeasts found on a specimen.

*L*_mean_10 logarithm of the mean number of viable yeasts found on the specimens.

A value ≤ 0.2 indicated a valid test result.

An additional test was implemented for *C. albicans* to test the survival of the *Candida* genus in dry environmental conditions (*C. auris* strains have not been evaluated with this test due to safety concerns). The methodology for the dry test was based on our own protocol (Poelzl et al., 2025). The fungal suspension was prepared as described above for the antimicrobial assay specified by ISO 22196:2011, but the application was conducted through the spray chamber CAMAG Derivatizer Base Unit (CAMAG® Derivatizer, Muttenz, Switzerland). In each spraying process, eight samples were simultaneously sprayed with a fungal suspension of 2.5 × 10^5^–10 × 10^5^ CFU/mL. After the process in the aerosol generating chamber, the samples were dried on a sterilized plastic rack. After drying, the 0 h samples were transferred into a plastic container containing 10 mL SCDLP medium and 12 g – 14 g glass beads (2.85–3.3 mm, Carl Roth GmbH + Co Kg, Karlsruhe, Germany) to rescue surviving *Candida*, with the test surface facing downwards. The other dried samples for further incubation (1 h and 24 h) were each transferred into a sterile petri dish (90 mm × 16.2 mm, Fisher Scientific, Schwerte, Germany) with the test surface facing upwards. The incubation was performed at 20 °C ± 1 °C with a RH between 30–65% for 1 h or 24 h. After the incubation these samples were also transferred into a plastic container with neutralizer and glass beads. The samples were shaken in the recovery liquid for 3 min with 200 rpm on a Battery Shaker KM 2 Akku (Edmund Bühler GmbH, Bodelshausen, Germany). The dilution series were performed in PBS. Finally, 500 μL of appropriate dilutions were plated on TSA plates in duplicates. All plates with 30–300 CFU after incubation for 48 h at 30 °C ± 2 °C were counted.

The applied load is defined as the actual number of yeast cells sprayed in the chamber and onto the eight specimens in the experiment. Time point 0 h is defined as the test point where the fungal suspension is completely dry on the surface and harvested immediately to ensure initial concentration of each sample. The entire process from the start of spraying in the chamber to the completion of surface drying, is typically completed within a time frame of 5 to 10 min, dependent on the specific surface condition. For each incubation time, duplicates (*n* = 2) of each sample type were used in two independent runs (*n* = 4) to calculate mean and standard deviation for the antifungal activity. When no colonies were countable on the plates, the limit of detection was set as 10 CFU, since 10 mL of the neutralization medium was used.

The same formulas as above have been used to calculate the results as well as the reduction and the verification of the method. An additional verification of the “dry-test” method was implemented (Poelzl et al., 2025) with following formula:


$$\frac{\left(X/2\right)}{S_{0}}\leq 0.5\;\log_{10}$$

*X*initial suspension concentration (applied load) in CFU (divided by 2, since only half of the samples (= 0 h) of one run were included).

*S*_0_sum of the common logarithm of the number of viable yeasts recovered from the test specimens immediately after inoculation (0 h).

The average of the two independent runs was calculated and a value ≤ 0.5 log_10_ usually indicates a low loss of the test organism and therefore a good initial concentration. However, preliminary experiments with *C. albicans* already demonstrated a loss greater than 0.5 log_10_, attributable to the diminished permeability of the nozzle for the larger eukaryotic cells (Poelzl et al., 2025). Furthermore, exceptions can be assumed for surfaces with a rapid antimicrobial efficacy (= AMC-TX-7770). Consequently, deviations from the specified value are to be anticipated.

### 2.4 Data analysis

Results were expressed as described with corresponding mean ± standard deviation (SD). Depictions were generated using CorelDRAW 2019 (Corel Corporation, Ottawa, Canada) and GraphPad Prism Version 10 (GraphPad Software, Boston, MA, United States).

Statistical analyzes were performed through GraphPad Prism Version 10 using Mann–Whitney U-test (mean with 95% CI, *p*-value: <0.05).

## 3 Results

**Figure 1:**
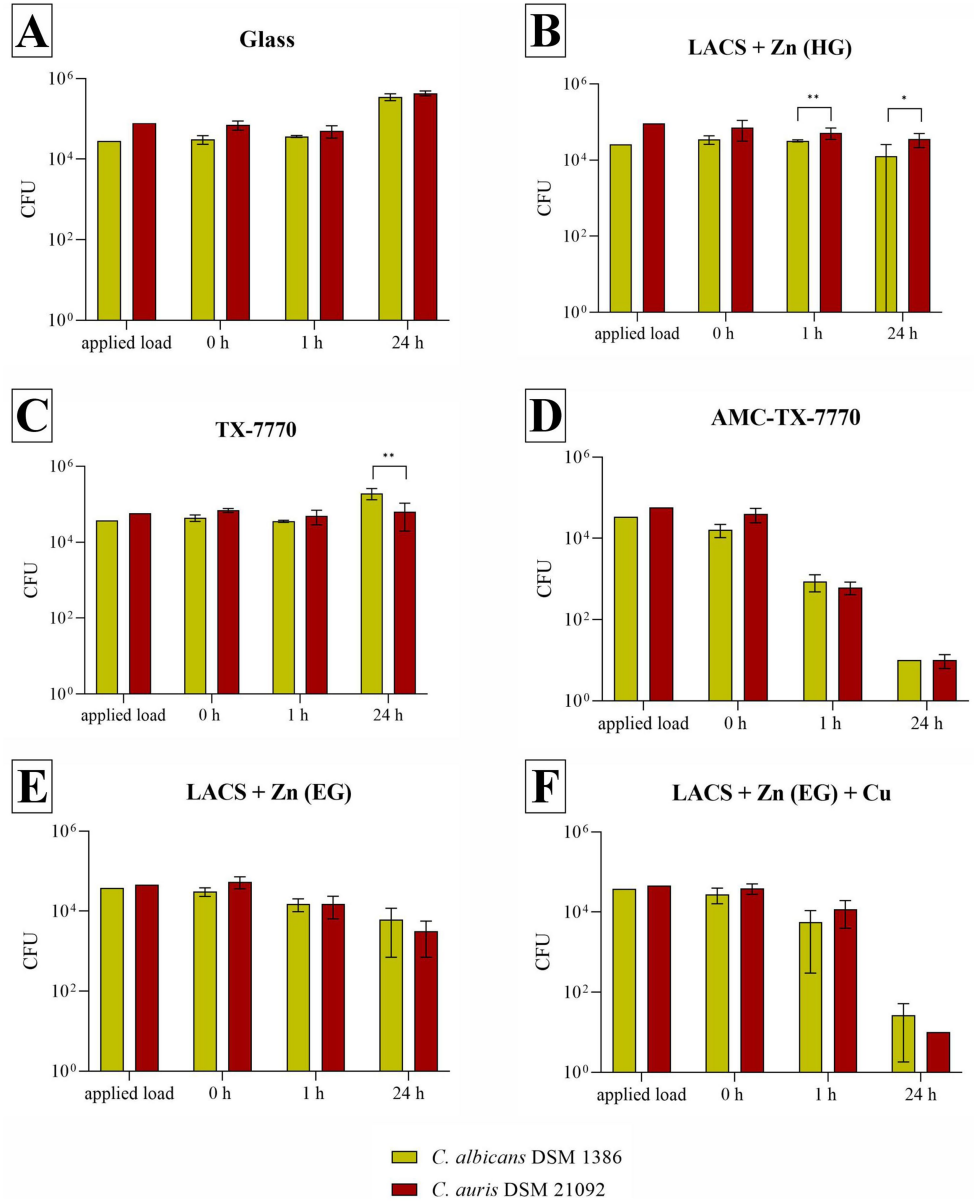
Quantification of *C. albicans* DSM 1386 and *C. auris* DSM 21092 after incubation on tested surfaces according to ISO 22196:2011. Specimens of Glass **(A)**, LACS + Zn (HG) **(B)**, TX-7770 **(C)**, AMC-TX-7770 **(D)**, LACS + Zn (EG) **(E)** and LACS + Zn (EG) + Cu **(F)** were incubated with a yeast concentration of 2.5 × 10^5^ – 10 × 10^5^ CFU/mL. The temporal incubations (1 h and 24 h) were performed at 30 °C ± 2 °C (*C. albicans*) or at 25 °C ± 2 °C (*C. auris*) and at a RH of >90%. Further, 0 h shows the recovery immediately after inoculation on the tested specimens. After the time points, the yeasts were harvested and checked for their survival. The error bars indicate the standard errors of the respective means, which were composed of triplicates in two independent runs (*n* = 6). The limit of detection was set as 10 CFU. Statistically significant differences between the two test organisms within the same incubation time are marked (mean with 95% Cl; Mann–Whitney U test; *p*-value: <0.05; * indicates statistical significance with a *p*-value below 0.033, ** indicates statistical significance with a *p*-value below 0.002).

The following experiments should demonstrate if the different surfaces show antifungal efficacy against the used DSMZ yeast strains (Figure 1). As expected, the overall reference Glass showed no effect. On the contrary, an increase of approximately 1 log_10_ was observed after 24 h for both fungal DSMZ strains. For the reference of the applied clear lacquer without any antimicrobial agents (TX-7770), also no efficacy could be determined as the reduction after the incubation time points was either < 0.5 log_10_ or an increase compared to the 0 h was evident (*C. albicans* after 24 h). *C. albicans* and *C. auris* could also be stably detected after 1 h and 24 h on the Zn-coated steel surfaces, regardless of whether they were coated through hot-dip galvanization (HG) or through electrolytic galvanization (EG). Therefore, antifungal activity could only be detected on AMC-TX-7770 and LACS + Zn (EG) + Cu. The reduction from the initial concentration (0 h) on these two surfaces after 24 h was > 3 log_10_ for both test organisms. AMC-TX-7770 showed the fastest fungal efficacy compared to all other samples, with a 1.2 log_10_ reduction for *C. albicans* and a 1.6 log_10_ reduction for *C. auris* after 1 h (Table 3).

**Table 3:** Calculated reduction of *C. albicans* DSM 1386 and *C. auris* DSM 21092 after the different incubation times tested on the six samples and test validity.

Sample	Time	*C. albicans* DSM 1386			*C. auris* DSM 21092		
		%	log_10_	Test validity	%	log_10_	Test validity
Glass	1 h	x	x	0.06	28.17	0.14	0.06
	24 h	x	x		x	x	
LACS + Zn (HG)	1 h	8.37	0.11	0.07	26.60	0.14	0.18
	24 h	63.46	0.27		50.05	0.20	
TX-7770	1 h	18.59	0.12	0.06	28.67	0.14	0.03
	24 h	x	x		8.46	0.11	
AMC-TX-7770	1 h	94.58	1.19	0.11	98.42	1.63	0.11
	24 h	99.94	3.16		99.97	3.40	
LACS + Zn (EG)	1 h	50.98	0.20	0.07	72.24	0.36	0.10
	24 h	79.68	0.49		94.12	1.17	
LACS + Zn (EG) + Cu	1 h	79.81	0.50	0.13	70.09	0.33	0.07
	24 h	99.90	3.01		99.97	3.39	

The decrease after 1 h and 24 h was calculated in percentage or log_10_ by the results of 0 h on each tested sample. x refers to those results where no reduction was calculable (i.e., increase in concentration compared to 0 h). For the results of the test validity all values (*n* = 6) of two independent runs were considered.

Furthermore, the experiments against the two DSMZ strains proved that *C. albicans* DSM 1386 achieved representative results for *C. auris* in this test setup. As shown in Figure 1 the results of the two test organisms are similar on all test surfaces. This was also confirmed by the Mann–Whitney U test, which was only able to calculate a significant difference between the strains on LACS + Zn (HG) at 1 h and 24 h (probably due to the different applied load) as well as after 24 h on TX-7770.

**Figure 2:**
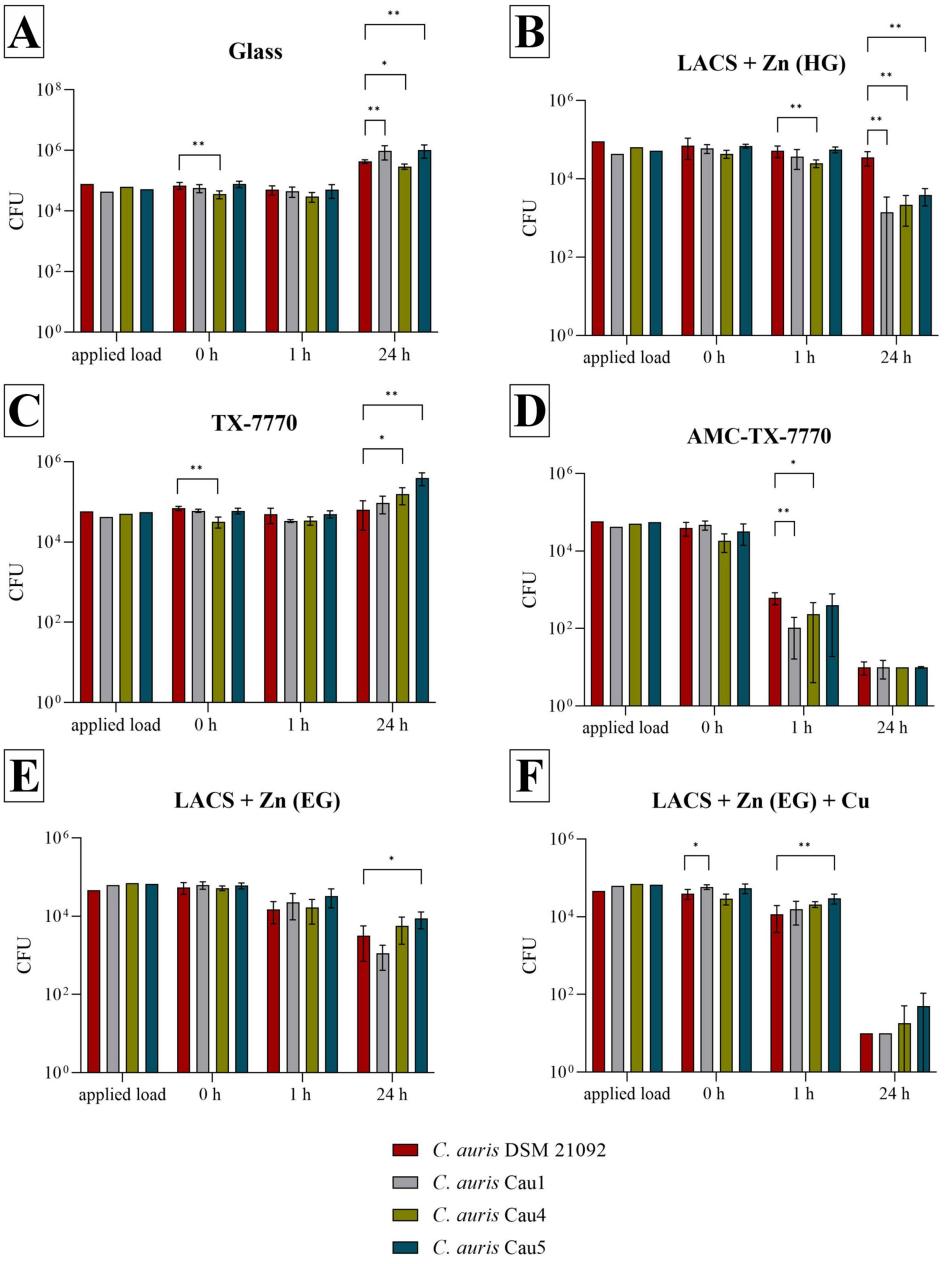
Survival of all tested *C. auris* strains after incubation on tested surfaces according to ISO 22196:2011. Specimens of Glass **(A)**, LACS + Zn (HG) **(B)**, TX-7770 **(C)**, AMC-TX-7770 **(D)**, LACS + Zn (EG) **(E)** and LACS + Zn (EG) + Cu **(F)** were incubated with a yeast concentration of 2.5 × 10^5^ – 10 × 10^5^ CFU/mL from *C. auris* DSM 21092 or with the clinical isolates Cau1, Cau4 or Cau5. The temporal incubations (1 h and 24 h) were performed at 25 °C ± 2 °C and at a RH of >90%. Further, 0 h shows the recovery immediately after inoculation on the tested specimens. After the time points, the yeasts were harvested and checked for their survival. The error bars indicate the standard errors of the respective means, which were composed of triplicates in two independent runs (*n* = 6). The limit of detection was set as 10 CFU. Statistically significant differences between the DSMZ strain and the clinical isolates within the same incubation time are marked (mean with 95% Cl; Mann–Whitney U test; *p*-value: <0.05; * indicates statistical significance with a *p*-value below 0.033, ** indicates statistical significance with a *p*-value below 0.002).

Further serial tests against the clinical isolates of *C. auris*, in comparison to the DSMZ strain of the same species, were performed to show whether the tested surfaces have a different effect on the tested strains. In comparison to the results of *C. albicans* DSM 1386 (Figure 1), more significant differences were calculable by means of the Mann–Whitney U test (Figure 2). In particular, significant differences were observed after 24 h of incubation on Glass and LACS + Zn (HG) between all three isolates to *C. auris* DSM 21092. However, the tendency of the surface efficacies is also clearly recognizable for the clinical isolates. As was the case with the DSMZ strain, fungal growth occurred on both the ultimate reference Glass and the additional reference TX-7770, when comparing the fungal concentration at 0 h and 24 h. Furthermore, no antifungal effect on LACS + Zn (EG) could be determined, as the reduction was 1.56 log_10_ for Cau1 or <1 log_10_ for *C. auris* DSM 21092 (Table 3), Cau4 and Cau5 (Table 4) after 24 h. The other Zn-coated surface (LACS + Zn (HG)) exhibited a reduction in the range of 1.18 log_10_ to 1.43 log_10_ for the isolates. Thus, a slight effect can be attributed to. In contrast, the same coating did not exert any influence on the DSMZ strain. A good antifungal activity with over 3 log_10_ was verifiable for AMC-TX-7770 and LACS + Zn (EG) + Cu. Nevertheless, as with the two DSMZ strains (Figure 1), the faster effect of the AMC-TX-7770 sample must be emphasized in this context. After 1 h of incubation on these surfaces, a marked reduction in fungal load was observed, from 1.79 log_10_ to 2.45 log_10_ (Table 4).

**Table 4:** Calculated reduction of the three *C. auris* isolates after the different incubation times tested on the six samples and test validity.

Sample	Time	*C. auris* Cau1			*C. auris* Cau4			*C. auris* Cau5		
		%	log_10_	Test validity	%	log_10_	Test validity	%	log_10_	Test validity
Glass	1 h	21.17	0.13	0.07	15.40	0.12	0.10	35.36	0.16	0.07
	24 h	x	x		x	x		x	x	
LACS + Zn (HG)	1 h	38.87	0.16	0.06	42.86	0.18	0.06	19.45	0.12	0.03
	24 h	97.68	1.43		94.96	1.20		94.45	1.18	
TX-7770	1 h	44.42	0.18	0.02	x	x	0.09	16.61	0.12	0.04
	24 h	x	x		x	x		x	x	
AMC- TX-7770	1 h	99.78	2.45	0.05	98.73	1.79	0.23	98.74	1.80	0.18
	24 h	99.98	3.47		99.95	3.19		99.97	3.32	
LACS + Zn (EG)	1 h	63.26	0.27	0.06	68.26	0.32	0.04	45.30	0.18	0.06
	24 h	98.22	1.56		89.01	0.91		85.46	0.69	
LACS + Zn (EG) + Cu	1 h	73.50	0.37	0.04	28.86	0.14	0.09	45.15	0.18	0.09
	24 h	99.98	3.58		99.94	3.16		99.91	3.11	

The decrease after 1 h and 24 h was calculated in percentage or log_10_ by the results of 0 h on each tested sample. x refers to those results where no reduction was calculable (i.e., increase in concentration compared to 0 h). For the results of the test validity all values (*n* = 6) of two independent runs were considered. A high variance was present between the individual runs of AMC-TX-7770 for Cau4, which is why the test validity value is also high. However, the individual runs had all values ≤ 0.2.

**Figure 3:**
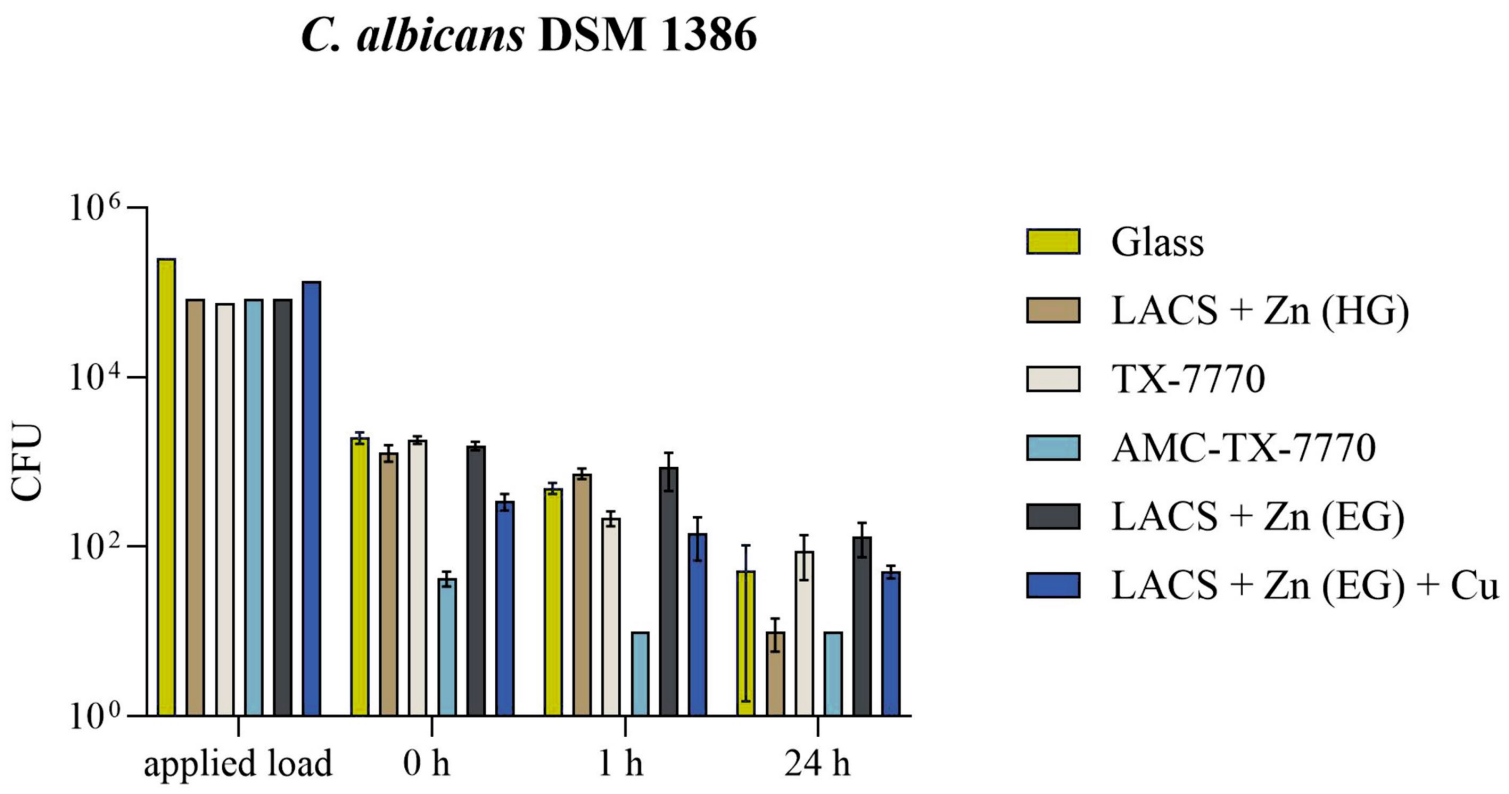
Survival of *C. albicans* DSM 1386 in a dry environment on the tested surfaces. Five hundred microlitre of fungal suspension with 2.5 × 10^5^ – 10 × 10^5^ CFU/mL were sprayed over eight samples in a CAMAG Derivatizer Base Unit, respectively. The temporal incubation (1 h and 24 h) was performed at 20 °C ± 1 °C and RH of 30–65%. Furthermore, 0 h shows recovery immediately after the suspension has dried on the tested surface of Glass, LACS + Zn (HG), TX-7770, AMC-TX-7770, LACS + Zn (EG) or LACS + Zn (EG) + Cu. After the time points, the yeasts were harvested and checked for survival. The error bars indicate the standard errors of the respective means, which were calculated from duplicates in two independent runs (*n* = 4). The limit of detection was 10 CFU.

Due to the comparability of the DSMZ strains in the standard ISO 22196:2011 test procedure, a dry test with the less pathogenic strain of *C. albicans* was performed to demonstrate the antifungal efficacy of the samples in a different test setup (Figure 3). In the dry test, AMC-TX-7770 again proved to be a very effective surface modification. Already after the drying time (approximately 5 min incubation on the samples) only a few colonies of the fungus could be detected and after 1 h *C. albicans* was completely eliminated. This efficacy also corresponds to the results of the “liquid-test” according to ISO 22196:2011, where the fastest effect was also observed on AMC-TX-7770 (Figures 1, 2). The other samples could not achieve this complete reduction in dry environmental conditions after 1 h as 2.2 × 10^2^ CFU to 8.7 × 10^2^ CFU were still detectable. A significant reduction of the initially sprayed fungal cells between 0 h and 1 h could be calculated on Glass, LACS + Zn (HG), TX-7770 and AMC-TX-7770. However, the reduction for all surfaces was less than 1 log_10_ within 1 h incubation period (0.18 to 0.84 log_10_, Table 5). After 24 h of incubation, not only AMC-TX-7770 (no colonies detectable), but also LACS + Zn (HG) showed an effect with a 2.13 log_10_ reduction compared to 0 h. All other surfaces achieved a minimal reduction of approximately 1 log_10_.

**Table 5:** Calculated reduction of *C. albicans* DSM 1386 in the dry environmental test after 1 h of incubation on the six samples, test validity and loss of applied load during the spraying process.

Sample	Time	*C. albicans* DSM 1386			
		%	log_10_	Test validity	Loss of applied load
Glass	1 h	74.51	0.39	0.06	1.32
	24 h	97.26	1.37		
LACS + Zn (HG)	1 h	43.41	0.18	0.09	0.82
	24 h	99.22	2.13		
TX-7770	1 h	88.03	0.84	0.03	0.52
	24 h	95.13	1.21		
AMC-TX-7770	1 h	76.47	0.43	0.14	2.25
	24 h	76.47	0.43		
LACS + Zn (EG)	1 h	43.85	0.18	0.04	0.69
	24 h	91.41	1.12		
LACS + Zn (EG) + Cu	1 h	57.58	0.24	0.11	1.49
	24 h	85.11	0.67		

The decrease after 1 h was calculated in percentage or log_10_ by the results of 0 h on each tested sample. For the results of the test validity all values (*n* = 4) of two independent runs were considered. The loss of fungal cells through the spraying process in the chamber was calculated for each independent run and the average value is shown.

## 4 Discussion

*Candidozyma auris* is a multidrug-resistant pathogen that has been classified as a serious health threat, particularly in healthcare settings. In order to prevent prolonged outbreaks in these vulnerable facilities in the future, all additional measures should be considered. Different strategies to eliminate *C. auris* have already been investigated, including classic antifungal agents, disinfectants, antiseptics and UV light therapy (Bandara and Samaranayake, 2022). In the light of emerging resistance against these conventional measures (Cadnum et al., 2017; Kean et al., 2018; Kean and Ramage, 2019), combinations of different decontamination strategies are imperative to ensure better containment of the problematic fungal pathogen. The use of antimicrobial surfaces is therefore another way to rapidly eliminate pathogenic microorganisms on frequently touched surfaces.

Therefore, this study investigated different common and specific surfaces for their antifungal efficacy against the *Candidozyma*/*Candida* genus. On common surfaces without antimicrobial additives such as glass, steel (LACS + Zn) or lacquer (TX-7770) no antifungal efficacy could be detected. Instead, an increase of the initial fungal concentration on Glass was evident for all tested strains after 24 h. Additional experiments according to ISO 22196:2011 with long-term testing up to three and a half weeks on the overall reference material Glass have also shown survival of *C. albicans* in our laboratory with no decrease from the initial concentration applied (publication in progress). These results prove the fungus’s capacity for persistence on ordinary surfaces, which was previously demonstrated in other studies showing survival for weeks (Welsh et al., 2017; Dire et al., 2023). However, investigations on surfaces with antifungal properties are limited, since tests against bacteria or viruses are preferred. Any antifungal surface tests have been carried out according to ISO 22196:2011, were conducted exclusively with *C. albicans* and even these tests are limited in scope. For instance, Scuri et al. tested two different silver additives against both standard test microorganisms (*Escherichia coli* and *Staphylococcus aureus*) and *C. albicans* and concluded that these surfaces also show antifungal properties and that ISO 22196:2011 can be extended to include fungal strains (Scuri et al., 2019). Other studies (Ghamrawi et al., 2017; Rehman et al., 2023) also employed *C. albicans* in accordance with ISO 22196:2011, thereby demonstrating antifungal activity on the surfaces tested. In the present study, *Candida* was also utilized for ISO 22196:2011, with no evident complications. Therefore, the incorporation of a standard fungal microorganism in antimicrobial surfaces testing is strongly advocated to enhance the testing capacity of surfaces against cells with eukaryotic structure. *C. albicans* would be a suitable choice as this fungus is already employed in disinfectant testing, for instance (Deutsche Gesellschaft Für Hygiene und Mikrobiologie, 2022). Müller et al. also confirmed the possibility of using *C. albicans* as a suitable surrogate test organism in EN 13624 and EN 16615, instead of testing against the highly pathogenic *C. auris* (Müller et al., 2020). The present study has now also confirmed the applicability of *C. albicans* as a model organism for *C. auris* on the selected surfaces, in accordance with the specific testing scenario of ISO 22196:2011. Analogous results were achieved on both, reference materials and surfaces with antimicrobial properties, for the DSMZ strains of *C. albicans* and *C. auris*. Although, *C. albicans* can be used as a suitable surrogate under the testing conditions of ISO 22196:2011, this may not be transferable to other testing scenarios or chemistries, e.g., disinfectants.

To date, the number of studies conducted with *C. auris* is limited to some antimicrobial complexes, including copper, zinc and silver. The efficacy of this complexes was analyzed independently from ISO 22196:2011. Zinc-chelating compounds showed antifungal effect against *C. auris* by transcriptomics, metal-addition experiments and mass spectrometric analyses (Shinohara et al., 2024). Further, a new copper-based complex, silver-based nanoparticle complexes and a nanoparticle combination of both metals were also validated with antifungal properties by different test strategies (Lara et al., 2020; Kamli et al., 2021; Frota et al., 2024; Jain et al., 2025). The study on the silver-copper nanoparticle complex also highlighted the enhanced antimicrobial properties compared to their monometallic counterparts. The synergistic effects between different metals in a coating are already known to improve efficacy (Garza-Cervantes et al., 2017; Poelzl et al., 2024) a phenomenon that has been corroborated in this study. The addition of a thin copper coating to the electrolytically galvanized low alloyed carbon steel (LACS + Zn (EG) + Cu) resulted in a pronounced effect with the desired antifungal effect of 3 log_10_ reduction for all strains tested after 24 h. Conversely, zinc alone (LACS + Zn (HG) and LACS + Zn (EG)), did not demonstrate any significant effect, with a reduction ranging from <1 log_10_ to a maximum of 1.6 log_10_. A faster antifungal effect could only be verified with AMC-TX-7770. This lacquer containing antimicrobial substances achieved an average reduction of 1.77 log_10_ after 1 h. Moreover, AMC-TX-7770 is the only surface modification that proved an effect under dry test conditions, where a decrease after the drying time (0 h) was evident and no colonies were detectable after 1 h of incubation on this lacquer. The difference in results between the two test strategies is not surprising, as the continuous wet conditions of the “liquid-test” (ISO 22196:2011) can increase the release of biocidal substances from the coating by diffusion (Bäumler et al., 2022). In addition, as described in the comparison of ISO 22196:2011 and ISO 7581:2023 (Maitz et al., 2024), the test conditions of the well-known ISO 22196:2011 are not realistic, because microorganisms in a liquid phase would not be normally distributed evenly over an inanimate surface area. Under real-world scenarios, the surface would dry out, assuming that the products are not in continuous contact with water or moisture. In addition, ISO 7581:2023 has also some disadvantages in implementation and reproducibility (e.g., applying and distribution of 1 μL suspensions). For these reasons, the new test variant with the aerosol generating chamber was also included in this study to assess the performance of the surfaces in dry environmental conditions. The problem with this concept, however, is the generation of aerosols, so tests with critical microorganisms such as *C. auris* have been avoided for safety reasons. Since the comparison of the DSMZ strains of *C. albicans* and *C. auris* in the “liquid-test” showed analogous results, it can be assumed, that a similar result can be expected in the “dry-test” as well. However, to be sure, a new reliable and reproducible dry-test protocol for *C. auris* would have to be established in the future. Another issue with testing *Candida* in the aerosol generating chamber is the diminished permeability of the nozzle for the larger eukaryotic cells, resulting in a greater than 0.5 log_10_ difference between the sprayed fungal suspension and the concentration applied on the eight surfaces tested simultaneously. In the absence of alternatives (e.g., higher permeability of the membrane), the only option would be to increase the initial concentration of the fungus in order to be able to spray higher concentrations in the chamber. However, as there is a risk of clogging the membrane and as the efficacy can still be assessed even with a low initial concentration at 0 h (between 4.3 × 10^1^ and 1.9 × 10^3^ CFU), we would advise against this option.

Testing of the three clinical isolates according to ISO 22196:2011 also demonstrated comparable efficacy to *C. auris* DSM 21092. Significant differences were calculated, but most showed a negligible variation of < 0.5 log_10_. Exceptions with higher variances were found for TX-7770 (Cau5, 24 h), AMC-TX-7770 (Cau1, 1 h) and especially for LACS + Zn (HG) for all isolates after 24 h. The impact of the surfaces on the different isolates was also very similar. No effect through different genetic characteristics and/or phylogeographic origins could be detected. However, with the DSMZ strain (first East Asian clade II), Cau1 (South Asian clade I), Cau4 (African clade III) and Cau5 (South Asian clade I), only three of the six confirmed clades have been tested (Khan et al., 2024; The Lancet Microbe, 2024). Therefore, further testing with other clades may be useful. Due to limited access to Austrian clinical isolates, this was not possible in this study (Spettel et al., 2023). Nevertheless, the results suggest that the antimicrobial surfaces are equally effective against multiple clades, which is important for their clinical application.

In conclusion, the study demonstrated that all surfaces tested exhibited equivalent effects on all fungal strains, including three different clades of *C. auris* and thereby validating the use of the less hazardous *C. albicans* DSM 1386 strain as a suitable surrogate test organism for the *Candidozyma/Candida* genus in accordance with ISO 22196:2011. This strain can also be utilized for the evaluation of surfaces under dry environmental conditions within an aerosol-generating chamber. Testing against the hazardous strain *C. auris* has not yet been performed due to safety concerns. Therefore, this study provides no definitive confirmation of the efficacy of the surface modifications against *Candidozyma* under dry conditions. In addition, it was demonstrated that *Candidozyma/Candida* exhibited minimal susceptibility to common surfaces, including glass, steel and lacquer, with the capacity to persist for the duration of the 24 h testing period. The findings further revealed that the combination of Zn and Cu coatings or antimicrobial compounds integrated in the lacquer led to the elimination of the fungi. Consequently, these two surface modifications can be regarded as effective tools for fighting *C. auris* in hygienically critical areas, but further testing is required for confirmation.

## Data Availability

The original contributions presented in the study are included in the article/supplementary material, further inquiries can be directed to the corresponding author.
